# Effect of 3`-Azido-3`-Deoxythymidine (AZT) on Telomerase Activity and Proliferation of HO-8910 Cell Line of Ovarian Cancer

**Published:** 2006-02

**Authors:** Hongmei Li, Tianbao Song, Weizhong Xu, Yuecheng Yu, Xiaoyan Xin, Du Hui

**Affiliations:** 1 *Key Laboratory of Environment and Genes Related to Diseases of Education Ministry, School of Medicine, Xi’an Jiaotong University, Xi’an, China;*; 2 *Department of Obstetrics and Gynecology, Xijing Hospital, Fourth Military Medical University, Xi’an, China*

**Keywords:** 3`-azido-3`-deoxythymidine (AZT), reverse transcriptase inhibitor, telomerase, proliferation, ovarian carcinoma cell

## Abstract

**Objective:**

To study the effect of 3`-azido-3`-deoxythymidine (AZT) on telomerase activity and the proliferation of ovarian cancer cells *in vitro*.

**Methods:**

Telomerase activity was detected by enzyme linked immunosorbent assay (ELISA) in treated and untreated HO-8910 cells by AZT. The detection of cell viability was performed with 3-(4,5-dimethylthiazol-2-yl)-2,5-Diphenyl tetrazolium bromide (MTT) assay and the ultrastructure of the cells was observed by electron microscopy. The apoptotic rate of the cells was measured by flow cytometry.

**Results:**

AZT significantly inhibited telomerase activity of HO-8910 cells, and the effect was both time- and dose-dependent. The HO-8910 cells treated at different concentrations of AZT showed a significant reduction of cell viability and morphological changes of apoptosis. The apoptotic peak was detected in the AZT treated cells and the apoptotic rate was 14.2%.

**Conclusion:**

AZT can effectively inhibit both telomerase activity and proliferation of human ovarian cancer HO-8910 cells *in vitro*, suggesting that AZT may be used in the clinic treatment of ovarian cancer.

## INTRODUCTION

Telomerase is a specialized reverse transcriptase (ribonucleoprotein polymerase) consisting of telomerase RNA and protein. Telomerase RNA with a short template element directs the synthesis of telemetric repeats at chromosome ends, maintains chromosomal stability, stabilizes telomere length, and leads to neoplasm occurrence and cell immortality (1, 2). The telomerase activity was expressed in 85% of human cancers but not or seldom in normal somatic cells and benign tumors (3, 4). Telomerase activation was thought to be an essential event for tumor proliferation (5, 6). 3`-azido-3`-deoxythymidine (AZT) belongs to nucleoside analogs, which is currently used in the treatment of acquired immunodeficiency syndrome (AIDS). The main effect of AZT is to inhibit the activity of reverse transcriptase and synthesis of virus (7, 8). In recent years, researchers have found that AZT can inhibit many enzyme activities of cells *in vitro*, especially the telomerase activity. AZT can also inhibit reverse transcription process, activity of reverse transcriptase, telomerase activity and telomere expanding. AZT can also inhibit cell division and telomere shortening. Thus cell stability is disrupted, and cell proliferation and growth are inhibited (9, 10).

Ovarian cancer is the most common malignant tumor of women, with high mortality and poor prognosis. Although advances in ovarian cancer treatment with cytoreductive surgery and chemotherapy have improved survival rates in the last decade, the prognosis remains poor in patients with ovarian carcinoma because of ineffective diagnosis and treatment. Therefore, it is essential to explore novel forms of diagnosis and treatment. Numerous studies have demonstrated that telomerase is highly expressed in ovarian cancer (11-13). In this study, the inhibitory effect of AZT on telomerase activity and proliferation of ovarian cancer cells *in vitro* were examined and the possibility of AZT for clinical therapy of ovarian cancer was explored.

## MATERIAL AND METHODS

### Regents

AZT was purchased from Sigma (St. Louis, MO, USA), telomerase ploymerase chain reaction-enzyme linked immunosorbent assay (PCR-ELISA) Kit from Boehringer Manheim (Germany), RPMI-1640 from Hyklong Company (USA), and trypsin from DIFCO Company (USA). Penicillin and streptomycin were the products of Pharmaceutical Factory of Haerbin, (China), fetal calf serum was obtained from Zhejiang Evergreen Company (China), 3-(4,5-dimethylthiazol-2-yl)-2,5-Diphenyl tetrazolium bromide (MTT) from Huamei Biological Company (China), and dimethyl sulfoxide from Jinshan Chemical Plant (Shanghai, China).

### Cell line

Ovarian cancer cell line HO-8910 was developed in the laboratory of the Department of Obstetrics and Gynecology, Xijing Hospital, China. HO-8910 cells were cultured in RPMI 1640 supplemented with 100mg/ml streptomycin, 100mg/ml penicillin and 10% fetal calf serum. The cells were incubated at 37°C in the presence of 5% CO_2_. Logarithmically growing cells were used for subsequent experiments.

### Telomerase activity assay

HO-8910 cells were randomly divided into experimental and the control groups. Cells in the experimental group were maintained in culture medium with supplemented with AZT at he concentrations of 0.5 mM, 0.8 mM, 1.0 mM and 1.5 mM for 24 h, 48 h and 72 h, respectively. The same culture medium without AZT was used for the cells in the control group. Cells were collected at different time points by centrifugation at 2000 g for 10 min at 4°C. The pelleted cells were resuspended in 200 μL lysis reagent and incubated on ice for 30 min followed by centrifugation at 12,000 g for 25 min at 4°C. In the control group, HO-8910 cells were treated for 10 min at 65°C. The telomerase activity of HO-8910 cells was measured using a telomerase PCR-ELISA Kit.

### MTT assay on suppression of tumor cell growth

HO-8910 cells were cultured in RPMI 1640 containing 10% fetal calf serum, in which the cultured cells reached over 90% confluence in 96 well plates. Different concentrations of AZT (0.05 mM, 0.1 mM, 0.2 mM, 0.5 mM, 0.8 mM, 1.0 mM, 1.2 mM, 1.25 mM, 1.6 mM, 2.0 mM) were added to the cells in the experimental group while equal volumes of culture medium were added to the cells in the control group. After 24, 48 and 72 h, 20 μL of MTT (5 mg/mL) was added to each well and the cells were incubated for another 4 h at 37°C. The supernatant was carefully removed, and 150 μL of dimethyl sulfoxide was added into each well and shaken for 10 min. Absorbance (A) of the samples was measured at 490 nm by an enzyme linked immunoassay meter. The inhibitory rate of tumor cells = (1 - A_AZT_/A_control_) ×100%.

### Ultrastructure of tumor cells

HO-8910 cells were cultured in 100 mL flasks in RPMI 1640 containing 10% fetal calf serum. When the cells reached over 90% confluence, AZT was added to the cells in the experimental group at the final concentration of 0.8 mM or 1.2 mM, and equal volume of culture medium was added to the cells in the control group. Subsequently, cells in both groups were incubated for another 72 h, after which cells were transferred to a fresh tube and collected by centrifugation at 2000 g for 10 min. 2 mL of 20 g/L glutaraldehyde was then added and the cells were fixed for 2 h. After cells were embedded and ultrathin sections were cut, the ultrastructure of the cells were observed by transmission electron microscope.

### Cell cycle definition

HO-8910 cells (5 × 10^5^) in the experimental group were added to culture medium containing 0.8 mM or 1.2 mM AZT and the cells in the control group were added to culture medium without AZT. After incubation for 72 h, the cells in both groups were collected by centrifugation at 1000 g for 10 min and the pellet was carefully washed twice with 0.01 M phosphate buffered saline (pH7.3). Then 1 mL of 70% ethanol was added to the collected cells and the cells were kept at 4°C for further processing. After washed twice with 0.01 M phosphate buffered saline, the cells were stained with 300 μL of propidium iodide for 30 min. The cell cycle was detected by flow cytometer.

### Statistical analysis

The SPSS 12.0 software was used to determine the statistical significance of the differences between the two groups. The result was examined by the Repeated Measure ANOVA. P value less than 0.05 was considered significant.

## RESULTS

### Inhibition of telomerase activity of HO-8910 cells by AZT

Telomerase activity of HO-8910 cells was inhibited by AZT at 0.5 mM for 24 h. The inhibitory effect was significantly increased with the elevation of drug concentration and elongation of exposure time. (*P*<0.05, Table 1).

**Table 1:** Inhibition of AZT on telomerase activity of HO-8910 cells

AZT Concentration (mM)	A _450nm_		
	24 h	48 h	72 h

Control	0.63 ± 0.5	0.58 ± 0.5	0.56 ± 0.7
0.5	0.49 ± 0.7^a^	0.46 ± 0.7^a^ ^b^	0.39 ± 0.6^a^ ^b^
0.8	0.45 ± 0.6^a^	0.40 ± 0.8^a^ ^b^	0.35 ± 0.6^a^ ^b^
1.0	0.34 ± 0.6^a^	0.29 ± 0.5^a^ ^b^	0.22 ± 0.4^a^ ^b^
1.5	0.30 ± 0.5^a^	0.28 ± 0.2^a^ ^b^	0.16 ± 0.3^a^ ^b^

a *P*<0.05, *vs* control group;

b *P*<0.05, *vs* value at 24 h.

### Suppression of cell growth with MTT assay

**Figure 1:**
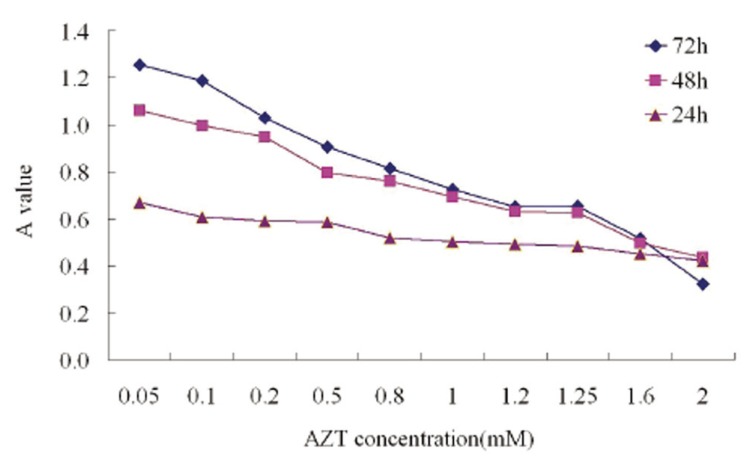
A value of HO-8910 cells exposed to AZT at different concentrations for 24 h, 48 h and 72 h.

The inhibition on HO-8910 cell growth of AZT at different concentrations for 24 h, 48 h and 72 h, respectively, was detected with MTT assay. The results showed that lower concentrations of AZT had a slight inhibitory effect on HO-8910 cells. With the increase of AZT concentration, the A value was decreased and the inhibitory rate was increased and the effect was dose-dependent. Similarly, with the elongation of acting time, the inhibitory rate was also increased in a time-dependent manner. The minimum inhibitory effect appeared with 0.8 mM at 24 h, with 0.5 mM AZT at 48 h, and with 0.1 mM AZT at 72 h (Fig. 1, Table 2).

**Table 2:** Inhibitory rate (%) of AZT on the growth of HO-8910 cells

Time (h)	AZT Concentrations (mM)									
	0.05	0.1	0.2	0.5	0.8	1.0	1.2	1.25	1.6	2.0

72	4	11	23	32	41	46	52	52	62	67
48	6	12	16	30	34	39	44	45	56	62
24	7	16	18	19	28	30	32	33	38	42

### Ultrastructural changes of HO-8910 cells

**Figure 2:**
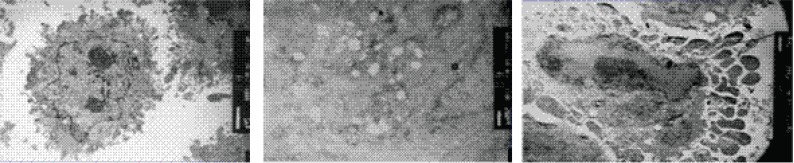
Morphologic changes of HO-8910 cells in the control group (A) and in the groups treated with AZT at 0.8 mM (B) and 1.2 mM (C) for 72 h.

The cells in the control group were observed to be polygon- or anomalous-shaped with various sizes by electron microscope. There were lots of long and thin microvilli on cellular surface (Fig. 2A). These cells had well-developed organelles, including mitochondria, rough endoplasmic reticulum, lysosomes and free ribosomes, etc. The nucleus was large with many karyokinesis, and abnormal nucleus mitosis was observed. After treated with AZT at 0.8 mM for 72 h, some HO-8910 cells became swollen with dilated endoplasmic reticulum and fewer mitochondria appeared when compared with the cells in the control group (Fig. 2B). After treated with AZT at 1.2 mM for 72 h, the microvilli of the cells were decreased in number and plenty of bubble-shaped convex appeared on cell surface. The chromatin was condensed and aggregated against the nuclear membrane, the structure of which was intact (Fig. 2C).

### Changes of cell cycle

**Figure 3:**
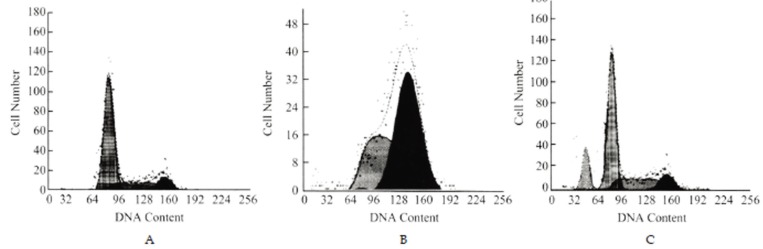
Cell cycles of HO-8910 cells in the control group (A) and in the groups treated with AZT at 0.8 mM (B) and 1.2 mM (C) for 72 h.

The cells treated with AZT at various concentrations had cell cycle profiles different from those of the cells in the control group. After exposure to 0.8 mM AZT for 72 h, the percentage of the cells in the phase G1 dropped from 57.6% (in control group) to 1.1%, the percentage of the phase G2/M cells rose from 15.5% to 57.6%, and the percentage of the phase S cells rose from 26.9% to 41.2%. When AZT was used at the concentration of 1.2 mM, the percentage of the phase G1cells rose to 67.7%, the phase G2 cells fell to 12.2%, and the phase S cells dropped to 20.1%. The apoptosis cusp was observed and the apoptotic rate was 14.2% in the group of 1.2 mM AZT (Fig. 3).

## DISCUSSIONS

AZT is a nucleoside analog able to inhibit reverse transcriptase by competing for deoxynucleotides, resulting in termination of chain elongation (1). AZT was originally used to treat AIDS. AZT not only inhibits certain enzyme activities of cells but also induces apoptosis of lymphoma correlated with AIDS (14, 15, 16). It is reported that AZT can inhibit telomerase activity and proliferation of mammary cancer cells, cervix cancer cells, and other cancer cells *in vitro* (17, 18).

In our study, we observed the effects of AZT at different concentrations on HO-8910 cells of ovarian cancer and their results at different time points. Our findings showed that AZT effectively inhibited telomerase activity and proliferation of human ovarian cancer HO-8910 cells *in vitro*, and that the viability of cancer cells had a significant reduction. At the same time points, the inhibitory effect of AZT was increased with the elevation of drug concentration; at the same drug concentration, its inhibitory effect was significantly increased with elongation of time. The inhibitory effect was both time- and dose-dependent. The notable cytotoxic effect of AZT on HO-8910 cells at 0.1 mM was observed 72 h after drug treatment and at 0.8 mM 24 h after AZT administration. The results indicated that drug concentration and exposure time of AZT were important for the suppressed growth of ovarian cancer HO-8910 cells. Cellular swelling, endoplasmic reticulum expanding and mitochondrium were observed to decrease in number by electron microscopy. The chromatin of cells was condensed and peripherally located. Flow cytometry detection showed that the cells treated with the drug at different concentrations had different cell cycles. The cells treated at low concentrations of AZT (0.8 mM) for 72 h were gathered at the phase G2/M. When the cells were treated with 1.2 mM of AZT for 72 h the cells at the phase G1 increased and the cells at the phase S decreased. The apoptotic peak was detected by flow cytometry and the apoptotic rate was 14.2%. The growth suppression of cells might be related to apoptosis. Nevertheless, it is still unknown whether AZT itself or apoptosis inhibiting genes induce the apoptosis, and the mechanism of cell apoptosis needs further investigation.

We also found that some cells survived at the 2.0 mM of AZT concentration due to their resistance to AZT. The development of these cells and their relation to drug resistance of tumor cells require further study.

Some researchers reported that the effect of tumor chemotherapy was more obvious when AZT was combined with drugs for chemotherapy. AZT may act as a synergist of drug for chemotherapy (19, 20). In our previous research we studied the effect of AZT combined with the drugs of chemotherapy on the growth of human ovarian cancer line HO-8910. The inhibitory effect on cancer cells of AZT combined with adriamycin or carboplatin was stronger than that of each drug singly used (21).

Chemotherapy is an essential therapeutic approach in the treatment of ovarian cancer. The results from our research showed that AZT had some significantly inhibitory effects on telomerase activity and proliferation of HO-8910 cell line of ovarian cancer cells. The ideal targeting strategy for tumor therapy should focus on some essential components present in tumor cells but not in normal cells. Telomerase is an essential condition of cell immortalization and an important factor of tumor development. Theoretically, the inhibition of telomerase activity by AZT may become a new treatment target because AZT can directly inhibit telomerase, which is absent in normal somatic cells. AZT can inhibit the growth of tumor cells with little injury to most normal cells by inhibiting telomerase activity. Moreover, it may also increase the specific effect and reduce the side effects of chemotherapy.

Since most tumor cells have telomerase activity, treating cancers by inhibiting telomerase activity is of great prospect (22). Therefore, the application of AZT might provide a novel approach to the targeted treatment of ovarian cancer and other cancers. However, the mechanism by which AZT inhibits the cell growth and interrupts the cell cycle of ovarian cancer needs further investigation.
